# Supplementary material for: Deep learning-enabled breast cancer hormonal receptor status determination from base-level H&E stains
Source: Nat Commun. 2020 Nov 16;11:5727. doi: 10.1038/s41467-020-19334-3 (PMC7670411; doi:10.1038/s41467-020-19334-3)
Supplement: Supplementary file 1 — Supplementary Information [file 41467_2020_19334_MOESM1_ESM.pdf]

Supplementary Information

Deep learning-enabled breast cancer hormonal receptor status determination from base-level H&E stains

Naik et al.

Supplementary Information

Deep learning-enabled breast cancer hormonal receptor status determination from base-level H&E stains

Nikhil Naik[1], Ali Madani[1#], Andre Esteva[1#], Nitish Shirish Keskar[1], Michael F. Press[2], Daniel Ruderman[3], David B. Agus[3], Richard Socher[1]

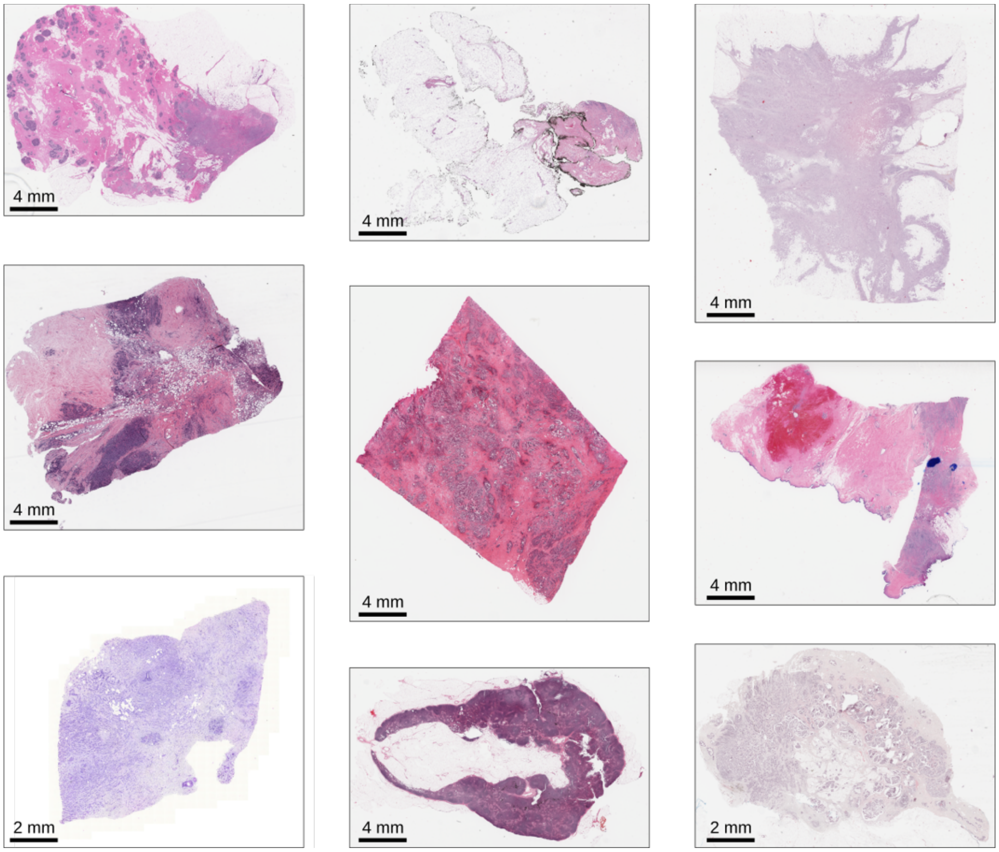

Supplementary Figure 1 | ReceptorNet is trained on a diverse, multi-country dataset obtained from the Australian Breast Cancer Tissue Bank (ABCTB) and The Cancer Genome Atlas (TCGA), with the TCGA samples obtained from 42 source sites from the USA, Poland, and Germany. The dataset has large variation in sample preparation, staining, and scanning quality.

---

1. Salesforce Research, 575 High St, Palo Alto, CA 94301. 2. Department of Pathology, Keck School of Medicine, University of Southern California, 2011 Zonal Ave, Los Angeles, CA 90033. 3. Lawrence J. Ellison Institute for Transformative Medicine, University of Southern California, 12414 Exposition Blvd Los Angeles, CA 90064. # - These authors contributed equally to the work. Corresponding author: Nikhil Naik (nnaik@salesforce.com).

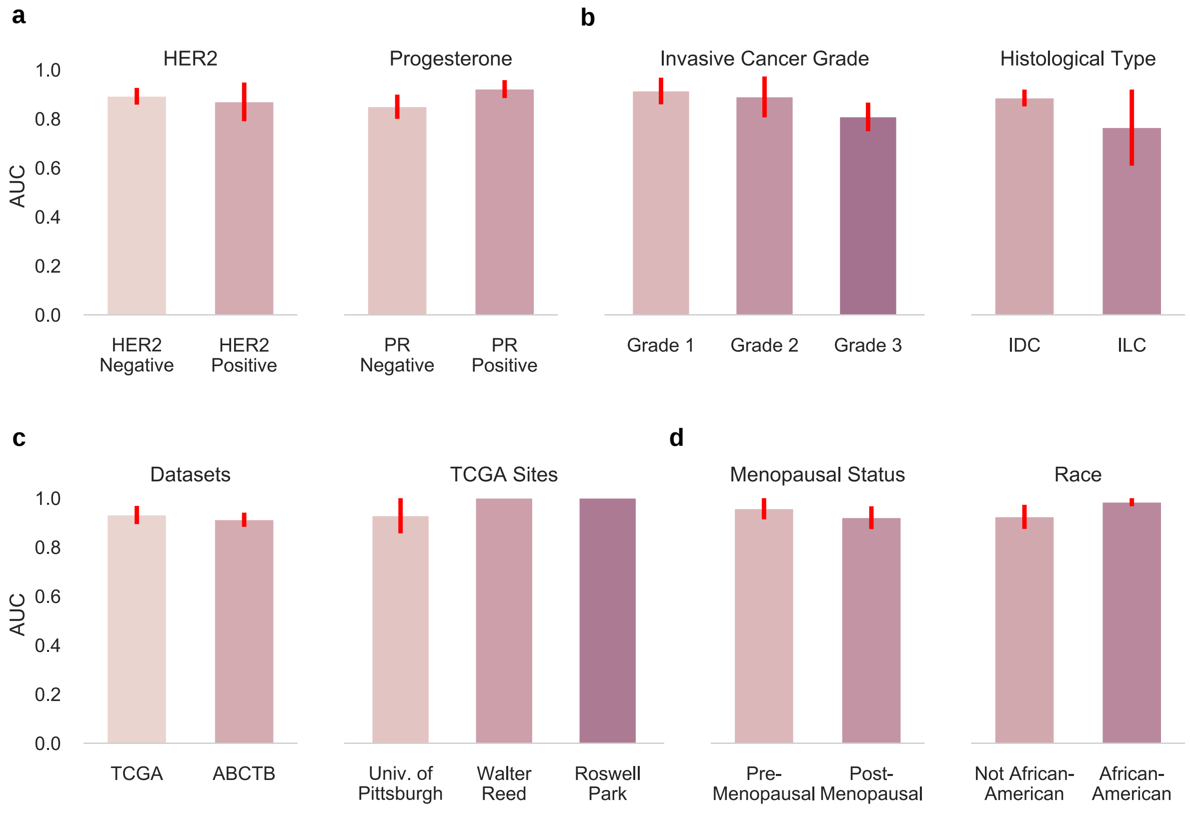

Supplementary Figure 2 | ReceptorNet performance across cohorts on the test set. We evaluate ReceptorNet performance across cohorts by splitting the data based on the presence or absence of other hormonal receptors (HER2-: N = 514, HER2+: N = 93; PR-: N = 210, PR+: N = 461) (**a**), tumor grade (Grade 1: N = 95, Grade 2: N = 194, Grade 3: N = 207), tumor origin location (IDC: N = 481 , ILC: N = 108) (**b**), source datasets (TCGA: N = 164, ABCTB: N = 507), tissue source sites (Univ. of Pittsburgh: N = 28, Walter Reed: N = 16, Roswell Park: N = 10) (**c**), and demographics (Pre-Menopausal: N = 49, Post-Menopausal: N = 115, Not African-American: N = 149, African-American: N = 15) (**d**). Performance trends are similar to the cross-validation of the train set (Fig. 2). Error bars represent 95% confidence interval for the true AUC calculated by bootstrapping the test set. Statistical tests for differences in AUC were performed using an upper tail F-test. Comparisons between different ERS prediction methods' AUCs on the same data set were performed using the DeLong method.

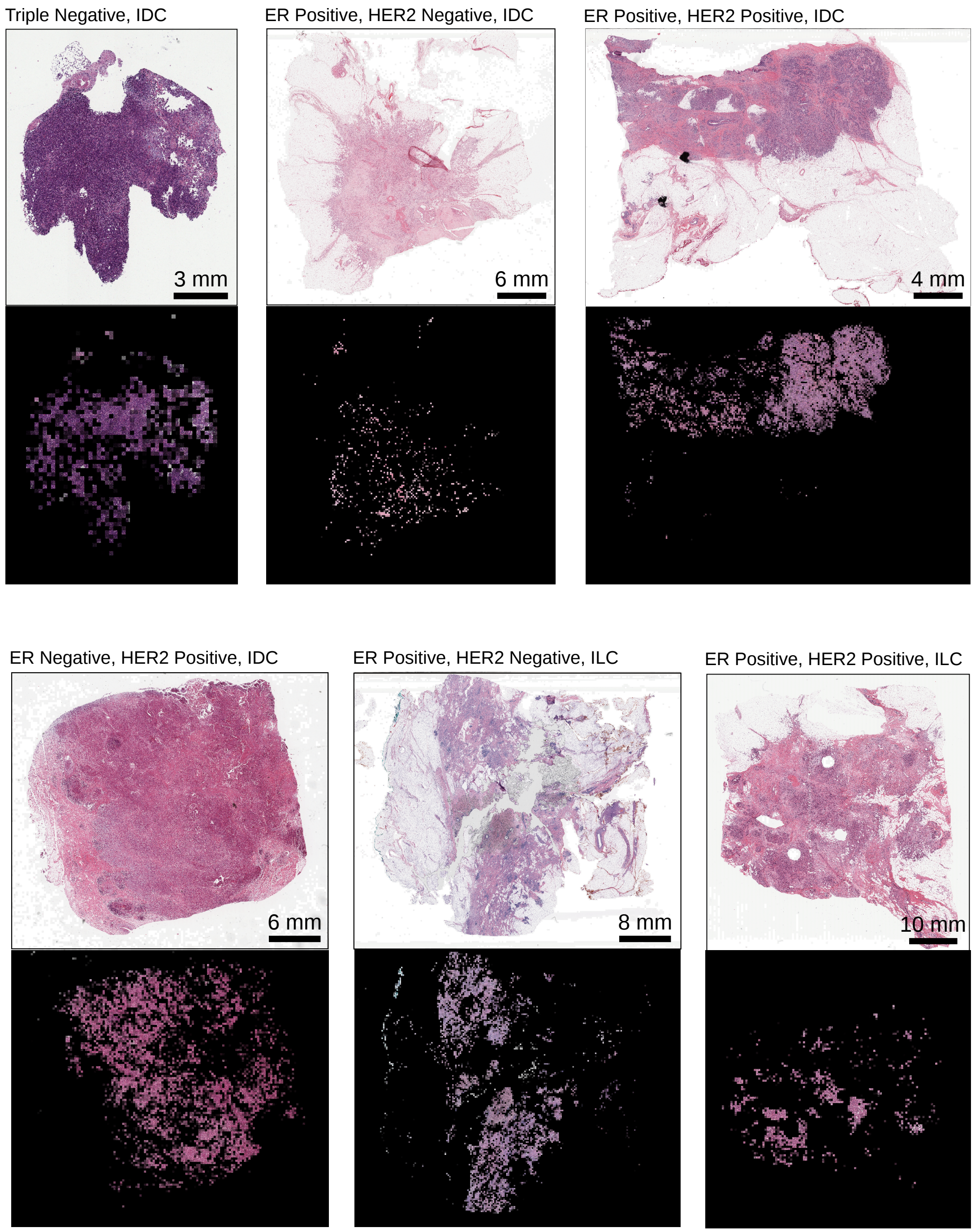

Supplementary Figure 3 | ReceptorNet identifies regions in WSI important for estrogen receptor status estimation. Using attention weights, we can visualize regions in whole slide images that are used by our algorithm for decision making. Here we show tiles with high attention weights.

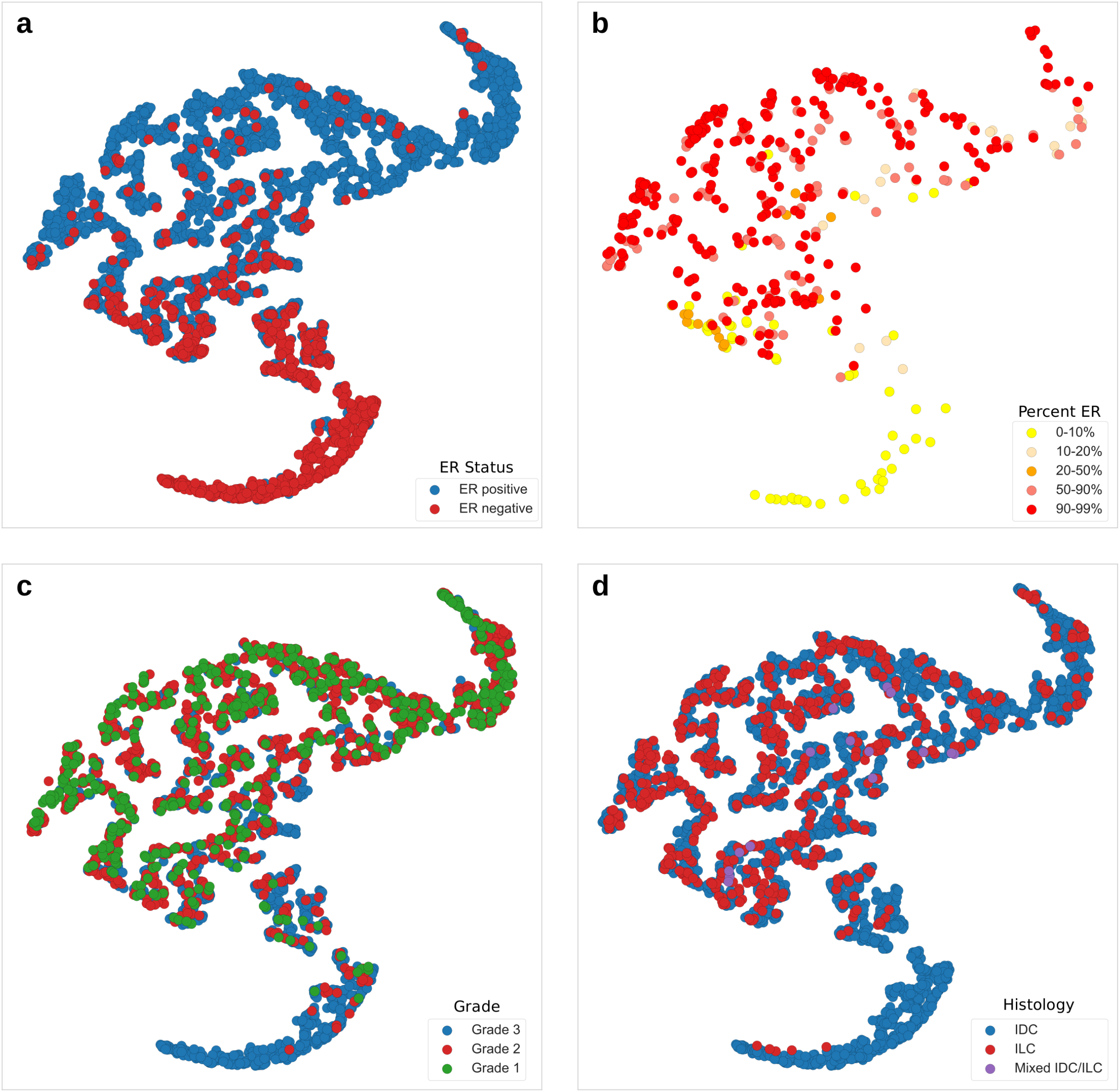

Supplementary Figure 4 | t-SNE visualization of the representation space of aggregate feature vectors of bag of tiles learnt by ReceptorNet. Two-dimensional t-SNE projection of the 512-dimensional representation space were generated from five randomly sampled bags of tiles per slide in the test set. Each data point is annotated using **a.** estrogen receptor status (n = 3525), **b.** percent estrogen receptor positive (n = 430), **c.** histological type (n = 3125), and **d.** tumor grade (n = 2495). n denotes the number of unique bags of tiles in the t-SNE plot. Percent estrogen receptor positive data was not available for ABCTB. Tumor grade was not available for TCGA.
