# Supplementary material for: Deep learning-enabled breast cancer hormonal receptor status determination from base-level H&E stains
Source: Nat Commun. 2020 Nov 16;11:5727. doi: 10.1038/s41467-020-19334-3 (PMC7670411; doi:10.1038/s41467-020-19334-3)
Supplement: Supplementary file 3 — Reporting Summary [file 41467_2020_19334_MOESM3_ESM.pdf]

nature research

Corresponding author(s): Nikhil Naik

Last updated by author(s): Sep 17, 2020

Reporting Summary

Nature Research wishes to improve the reproducibility of the work that we publish. This form provides structure for consistency and transparency in reporting. For further information on Nature Research policies, see Authors & Referees and the Editorial Policy Checklist .

Statistics

For all statistical analyses, confirm that the following items are present in the figure legend, table legend, main text, or Methods section.

n/a	Confirmed	
	x	The exact sample size (*n*) for each experimental group/condition, given as a discrete number and unit of measurement
x		A statement on whether measurements were taken from distinct samples or whether the same sample was measured repeatedly
x		The statistical test(s) used AND whether they are one- or two-sided<br>*Only common tests should be described solely by name; describe more complex techniques in the Methods section.*
	x	A description of all covariates tested
x		A description of any assumptions or corrections, such as tests of normality and adjustment for multiple comparisons
	x	A full description of the statistical parameters including central tendency (e.g. means) or other basic estimates (e.g. regression coefficient) AND variation (e.g. standard deviation) or associated estimates of uncertainty (e.g. confidence intervals)
x		For null hypothesis testing, the test statistic (e.g. *F*, *t*, *r*) with confidence intervals, effect sizes, degrees of freedom and *P* value noted<br>*Give P values as exact values whenever suitable.*
x		For Bayesian analysis, information on the choice of priors and Markov chain Monte Carlo settings
x		For hierarchical and complex designs, identification of the appropriate level for tests and full reporting of outcomes
	x	Estimates of effect sizes (e.g. Cohen's *d*, Pearson's *r*), indicating how they were calculated

Our web collection on statistics for biologists contains articles on many of the points above.

Software and code

Policy information about availability of computer code

Data collection	No software was used
Data analysis	Jupyter 4.4.0<br>Python 3.6.4<br>matplotlib==2.1.2<br>numpy==1.15.4<br>pytorch==0.4.1<br>scipy==1.0.0<br>openslide==3.4.1<br>pandas==0.22.0<br>scikit-learn==0.20.0<br>R==3.6.1

For manuscripts utilizing custom algorithms or software that are central to the research but not yet described in published literature, software must be made available to editors/reviewers. We strongly encourage code deposition in a community repository (e.g. GitHub). See the Nature Research guidelines for submitting code & software for further information.

Data

Policy information about availability of data

All manuscripts must include a data availability statement. This statement should provide the following information, where applicable:

- Accession codes, unique identifiers, or web links for publicly available datasets
- A list of figures that have associated raw data
- A description of any restrictions on data availability

TCGA dataset is publicly available at the TCGA portal (https://portal.gdc.cancer.gov). The ABCTB dataset is available from the Australian Breast Cancer Tissue Bank subject to ethical and scientific approvals as described in their access policy (https://abctb.org.au/abctbNew2/ACCESSPOLICY.pdf) The ImageNet dataset is publicly available at http://image-net.org/download. ImageNet-pretrained models in PyTorch can be accessed from https://pytorch.org/docs/stable/torchvision/models.html

Field-specific reporting

Please select the one below that is the best fit for your research. If you are not sure, read the appropriate sections before making your selection.

- [x] Life sciences
- [ ] Behavioural & social sciences
- [ ] Ecological, evolutionary & environmental sciences

For a reference copy of the document with all sections, see nature.com/documents/nr-reporting-summary-flat.pdf

Life sciences study design

All studies must disclose on these points even when the disclosure is negative.

Sample size	Unlike a hypothesis test, for which certain assumptions about effect size and variability imply a required sample size, in the current study we designed an algorithm using training data. The algorithm was then validated using an independent data set, verifying adequate training sample size.
Data exclusions	75 whole slide images were excluded because of excessive pen ink marks present on the image. The exclusion criteria was pre-established.
Replication	The results have been generated via a python codebase to guarantee that they can be reproduced easily. Experiments with a randomization part were performed multiple times to assess the reproducibility of the results.
Randomization	Randomization of patients for cross-validation was performed completely at random without any stratification.
Blinding	When performing experiments, blinding of experimenters to treatments helps to eliminate bias. Because no treatments were applied in this study, there was no need for blinding.

Reporting for specific materials, systems and methods

We require information from authors about some types of materials, experimental systems and methods used in many studies. Here, indicate whether each material, system or method listed is relevant to your study. If you are not sure if a list item applies to your research, read the appropriate section before selecting a response.

Materials & experimental systems

n/a	Involved in the study	
x		Antibodies
x		Eukaryotic cell lines
x		Palaeontology
x		Animals and other organisms
	x	Human research participants
x		Clinical data

Methods

n/a	Involved in the study	
x		ChIP-seq
x		Flow cytometry
x		MRI-based neuroimaging

Human research participants

Policy information about studies involving human research participants

Population characteristics	Digital images of microscope slides from patients that were diagnosed at various centers. Data obtained from the cancer genomic atlas and the Australian breast cancer tissue bank.
Recruitment	No patient recruitment was performed.
Ethics oversight	University of Southern California

Note that full information on the approval of the study protocol must also be provided in the manuscript.
